# The effects of physical activity on executive function in preschool children: a meta-analysis of randomized controlled trials

**DOI:** 10.3389/fpsyg.2026.1882118

**Published:** 2026-07-17

**Authors:** Wanxu Liu, Zilin Wang, Jingyao Yi, Xiaofen Li

**Affiliations:** 1 School of the Arts, Beijing Sport University, Beijing, China; 2 School of Sport Science, Beijing Sport University, Beijing, China

**Keywords:** executive function, meta-analysis, physical activity, preschool children, randomized controlled trial

## Abstract

**Objectives:**

Drawing on perspectives from embodied cognition research, this study systematically integrated high-quality randomized controlled trials to provide the first comprehensive evaluation of physical activity (PA) effects on the different dimensions of executive functions (EFs) in preschool children aged 2–6 years.

**Methods:**

Seven electronic databases were used to search for potential studies from inception until October 2025. Randomized controlled trials (RCTs) investigating the effect of PA on EFs in preschool children compared to a control condition were included. The effect sizes were synthesized using a random effects model with a 95% confidence interval.

**Results:**

We identified 31 RCTs (34 interventions; 2,330 preschoolers) through a systematic search. PA produced small-to-moderate benefits across all EFs: strongest effects on inhibitory control (IC) (SMD = 0.42, *p* < 0.001), followed by working memory (WM) (SMD = 0.30, *p* < 0.001) and cognitive flexibility (CF) (SMD = 0.25, *p* < 0.001). Subgroup analyses revealed that game-based interventions were particularly effective for CF (SMD = 0.54 vs. 0.11, *p* = 0.011), while cognitively engaging interventions showed greater benefits for WM (SMD = 0.55 vs. 0.06, *p* = 0.006). Session duration significantly moderated effects, with sessions ≥45 min yielding the largest benefits for CF (SMD = 0.61) and sessions ≥40 min for IC (SMD = 0.70). Additionally, interventions using passive control groups consistently demonstrated larger effect sizes than those with active controls for IC and WM (*p* < 0.001).

**Conclusion:**

PA positively influences all core EFs in preschoolers, with the most substantial benefits observed for IC. To maximize cognitive outcomes, PA programs should incorporate game-based elements, include cognitive challenges—particularly for WM and CF—and ensure session durations of approximately 45 min. These evidence-based recommendations provide practical guidance for designing effective PA interventions to support cognitive development in early childhood.

**Systematic review registration:**

https://www.crd.york.ac.uk/PROSPERO/view/CRD42022351779, Identifier: CRD42022351779.

## 1 Introduction

Executive functions (EFs), as a higher-order cognitive process that regulates goal-directed behavior, consists of three core components: cognitive flexibility (CF), inhibitory control (IC), and working memory (WM) (Diamond, 2012). The preschool period represents a critical window for the development of EFs. It not only directly influences children’ s language learning and social interactions, but also has long-term impacts on intellectual growth, emotional regulation, and the shaping of social behaviors (Doebel et al., 2016; Micalizzi et al., 2019; Veraksa et al., 2018; Zelazo et al., 2016).

According to embodied cognition theory, physical activity (PA) can promote cognitive development through gestures, object manipulation, and whole-body activities (Guare, 2014; Zou et al., 2025). Recent evidence suggests that PA enhances EFs through at least two complementary pathways among athers: an aerobic–metabolic pathway involving increased cerebral blood flow, neurotrophic factor release (e.g., BDNF), and neurotransmitter modulation, and a cognitive–coordinative pathway that engages prefrontal cortex activation through complex motor skill learning and executive demands during movement (Cabral et al., 2025; Singh et al., 2025; Voss et al., 2013). However, translating these mechanistic insights into consistent empirical outcomes has been challenging. Evidence from existing randomized controlled trials (RCTs) remains markedly heterogeneous: some studies report positive effects, such as improvements in WM following coordination training, while others find no significant benefits from programs like yoga or fundamental movement training (Aleksić Veljković et al., 2021; Lai et al., 2020; Lee et al., 2020). This empirical inconsistency suggests that the relationship between PA and EFs is not straightforward, but likely moderated by other factors. Consequently, the critical question has evolved from whether PA enhances EFs to under what conditions—and through what specific components—its benefits are most reliably realized. To address this question and account for the variability introduced by differences in assessment tools and sample characteristics, a meta-analytic approach becomes necessary to systematically quantify overall effects and identify the key moderators determining intervention success.

Although systematic reviews have examined the relationship between PA and children’ s cognitive development, meta-analyses focusing specifically on EFs during the preschool stage remain limited. Existing studies have predominantly focused on school-aged children or adolescents, neglecting the preschool period as a critical developmental window (de Greeff et al., 2018; Yan Jin et al., 2020). Several recent meta-analyses have synthesized evidence on PA and cognitive outcomes in young children, yet each has left important gaps: some have focused on broader cognitive outcomes without differentiating among EF subdomains (Wei et al., 2024), others have targeted clinical populations (Song Y. et al., 2023) or school-aged children (Cabral et al., 2025) rather than typically developing preschoolers, and even Morales et al. (2024)—despite reporting subdomain-specific effects—did not examine intervention design features such as game-based versus non-game-based delivery or control group type as moderators. Moreover, several studies have examined only one subcomponent of EFs, such as CF (Lage et al., 2024), IC (Dhir et al., 2021), or WM (Jun, 2018), and, given the relatively small number of included publications, the robustness of these findings remains uncertain. Finally, most moderator analyses in existing research are limited to factors such as acute exercise effects (Li et al., 2020), exercise intensity levels (Hu et al., 2021; Xie Chao et al., 2017), or comparisons between acute and chronic PA (Liu et al., 2020). Far fewer studies have systematically examined intervention types (e.g., free play vs. structured activities) or characteristics (e.g., duration and frequency), limiting the precision with which interventions can be implemented.

By synthesizing high-quality RCTs, the present study is the first to systematically evaluate the effects of PA on different dimensions of EFs in preschool children aged 2–6 years. Compared with previous research, this study makes several novel contributions: (1) it strictly restricts inclusion to RCTs to enhance the strength of causal inference; (2) it specifically targets the preschool period; (3) it differentiates among the three subcomponents of EFs for refined analysis; and (4) it comprehensively examines the moderating effects of different types and characteristics of PA interventions. The findings are expected to not only provide scientific evidence for preschool education practice but also lay a theoretical foundation for the development of evidence-based PA interventions for children.

## 2 Methods

### 2.1 Search strategy

A systematic search was conducted in China national knowledge infrastructure, Wanfang data, VIP databases, PubMed, Web of Science Core Collection, Cochrane Library, and the EBSCO self-built database to identify all relevant studies published up to October 20, 2025. In each database, the following groups of search terms were combined to identify potential studies: (1) “physical activity” OR “activity” OR “physical education” OR “exercise” OR “sport” OR “active games” OR “active play” OR “physical fitness” OR “music” OR “dance”; (2) “kindergarten” OR “Preschool” OR “young children” OR “early childhood”; (3) “executive function” OR “working memory” OR “inhibitory control” OR “cognitive flexibility” OR “cognition” OR “cognitive function.” The complete search strategies are provided in Supplementary File 1. The current study followed the PRISMA (Preferred Reporting Items for Systematic Reviews and Meta-Analyses) guidelines and was prospectively registered in PROSPERO (CRD42022351779; https://www.crd.york.ac.uk/PROSPERO/view/CRD42022351779).

### 2.2 Inclusion and exclusion criteria

Following the PICOS framework, studies were screened based on the following inclusion criteria: (1) participants were healthy preschool children aged 2–6 years without diagnosed diseases; (2) interventions involved PA in the form of exercise, structured training programs, and active games; (3) control groups engaged in sedentary activities such as art or cognitive tasks, or served as blank controls, free play, or regular curricula; (4) outcomes included at least one measure of EFs subdomains; (5) study design was a randomized controlled trial (RCT); (6) studies were published in Chinese or English; (7) only full-text articles available from core journals were included, and in cases of duplicate publications, only the most complete dataset was retained.

The exclusion criteria for the meta-analysis were as follows: (1) studies involving participants with mental or cognitive disorders, neurological diseases, brain injuries, or physical conditions that could hinder participation in PA; (2) intervention programs for the experimental group that included prolonged sedentary activities or non-PA formats; (3) control group interventions that involved dance, exercise, or other forms of PA; (4) studies that assessed only overall EFs or other cognitive domains, or those with incomplete or missing data on intervention outcomes; (5) studies that did not explicitly identify their design as an RCT.

### 2.3 Data extraction and quality assessment

Two researchers independently screened the retrieved records, first removing duplicates and then performing an initial screening based on titles and abstracts. Full-text articles were subsequently assessed for eligibility to determine whether they met the inclusion criteria. Any disagreements were resolved through discussion with a third researcher. Data were extracted independently by two researchers using a self-designed extraction form. Data were extracted using a self-designed form covering: (1) study characteristics (first author, year of publication); (2) participant and intervention details (sample size, age, intervention features, Details of intervention measures); (3) EFs assessment tools. In case of any disagreement during the extraction process, it should be resolved through joint review of the original text and discussion, with a third researcher consulted when necessary.

For subsequent subgroup analyses, interventions were categorized into the following groups: (1) high vs. low cognitive engagement, based on whether the intervention protocol explicitly targeted EF with the aim of promoting EF and cognitive development; (2) game-based vs. non–game-based interventions, according to whether the primary intervention activities were delivered in a game format; and (3) passive vs. active control groups. Intervention characteristics were further classified by session duration, frequency, and intervention period. In addition, only task-based measures of EF subcomponents—CF, IC, and WM—were included in this study, while overall EF composite scores and questionnaire-based measures were excluded. For studies employing mid-test designs, outcomes were separated into pretest–midtest and pretest–posttest comparisons for analysis. Studies that included multiple experimental groups involving physical activity were treated as separate comparisons, with each experimental group analyzed against the shared control group independently.

The methodological quality of included studies was assessed using the Cochrane Collaboration’ s Risk of Bias tool, which evaluates seven domains: (1) generation of random sequence; (2) allocation concealment; (3) blinding of participants and personnel; (4) blinding of outcome assessors; (5) completeness of outcome data; (6) selective reporting of outcomes; and (7) other potential sources of bias. For the purpose of analysis, studies with five or more domains rated as low risk were classified as high-quality, those with three to four low-risk domains as moderate-quality, and those with fewer than three low-risk domains as low-quality.

### 2.4 Statistical analysis

Statistical analyses were conducted using Review Manager 5.4.1 and Stata 15. If effect sizes in the included studies were reported in formats such as SE or 95% CI, we converted them into Mean ± SD to ensure consistency. Because different studies used various measurement tools for the same outcomes, standardized mean differences (SMD, Cohen’ s d) were used to quantify effect sizes between the intervention and control groups. For the meta-analysis, the mean changes from pre- to post-intervention in both the experimental and control groups were calculated and used to compute effect sizes in Stata 15. The magnitude of Hedges’ g was interpreted using Cohen’ s guidelines, which distinguished between small (<0.2), moderate (0.5), and large (>0.8) effect sizes (Cohen, 1988). The positive effect size favored the intervention group, whereas the negative effect size favored the control group.

Heterogeneity was assessed using the *I*^2^ and Q statistics. *I*^2^ values of 25, 50, and 75% were defined as small, moderate, and large amounts of heterogeneity, respectively. A random-effects approach was used based on the assumption of different true effect sizes (Higgins and Thompson, 2002). Subgroup analyses were further conducted to explore potential sources of heterogeneity across studies. To further examine the continuous variables identified as significant in subgroup analyses, we conducted a meta-regression in Stata using the metareg command to assess their associations with effect sizes. Risk of bias assessment charts were generated with RevMan software (version 5.4). Stata software (version 15.0) was employed to generate forest and funnel plots, conduct sensitivity analyses, and visually examine potential publication bias. Forest plots were used to display individual study effect sizes with 95% confidence intervals and pooled estimates. The presence of a publication bias for CF, IC, and WM was assessed using a funnel plot and by performing Egger’ s linear regression method. If the Egger’ s test yielded a *p*-value > 0.05, the trim-and-fill method was subsequently applied to identify potentially missing studies and calculate the adjusted SMD. A *p*-value > 0.05 after adjustment indicates that publication bias may need to be considered when interpreting the results. Sensitivity analyses were performed using a random-effects model, by sequentially removing one study at a time from the pooled analysis. Statistical significance was set at *p* < 0.05 for all tests.

## 3 Results

### 3.1 Search result

**Figure 1:**
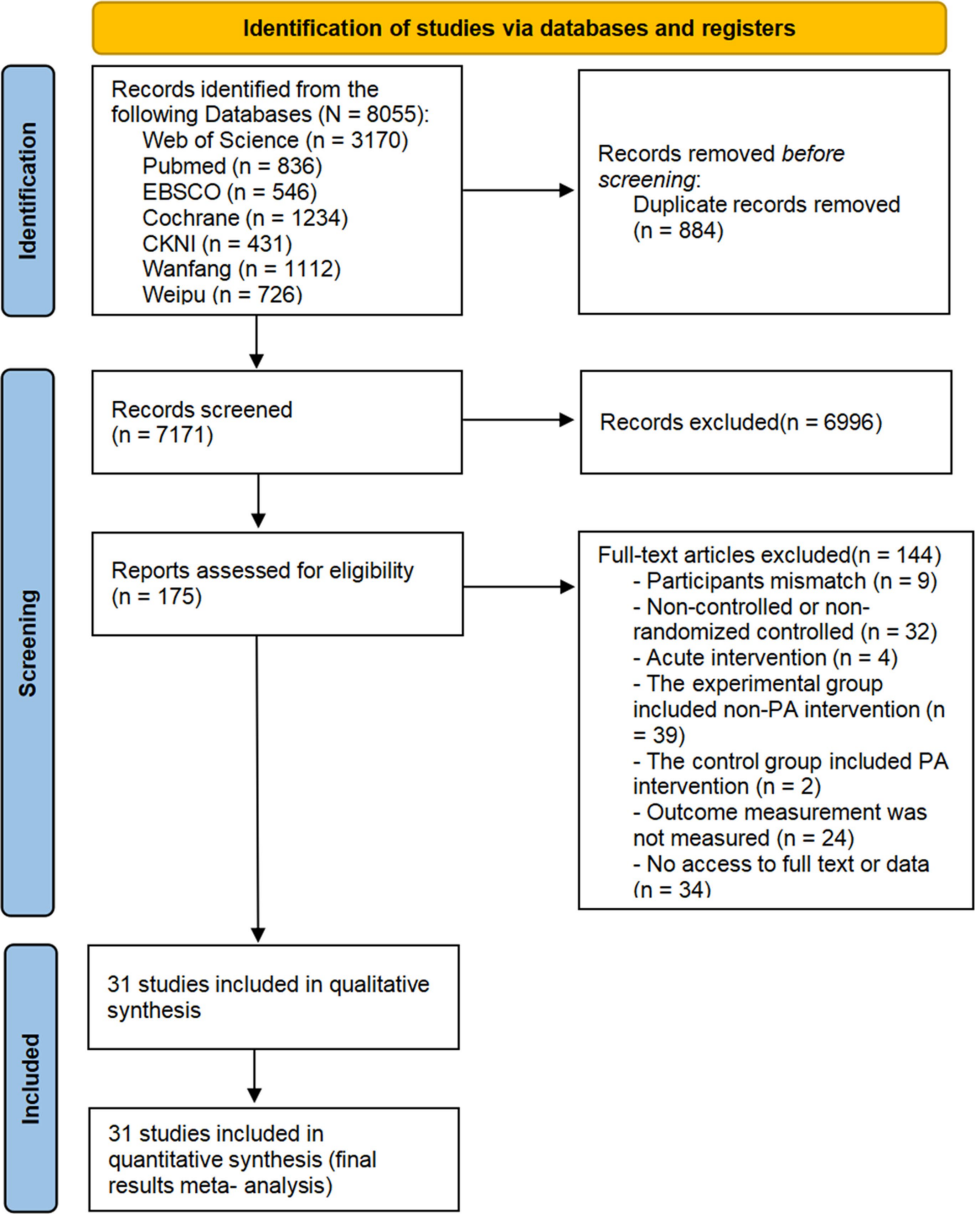
Flow diagram of each stage of the study selection.

Based on the aforementioned criteria, a total of 8,055 records were identified. After removing duplicates (*n* = 884), 7,171 records remained for further screening. Subsequently, 6,996 records were excluded following title and abstract screening. After full-text review, 144 articles were excluded, resulting in 31 randomized controlled trials (RCTs) meeting the inclusion criteria for this meta-analysis. The study selection process is illustrated in Figure 1 (n indicates the number of studies at each stage).

### 3.2 Characteristics of included studies

The studies included in the meta-analysis were coded for the following information: study details (author and year of publication), country, participants’ age, sample size, intervention, control condition, intervention frequency, intervention duration, intervention effects, and outcome measures. A total of 31 articles, comprising 34 independent datasets, were included in this meta-analysis, with 5 published in Chinese and 26 in English. The combined sample consisted of 2,330 participants, of whom 1,243 were assigned to intervention groups and 1,107 to control groups. Sample sizes across individual studies ranged from 20 to 201. The PA interventions varied widely, encompassing exercise (e.g., running, swimming, soccer), structured training programs (e.g., gymnastics, taekwondo, dance), and active games (e.g., rule-based gamified activities). Intervention durations ranged from 4 to 16 weeks, with session times ranging from 15 to 60 min and frequencies of 2 to 5 sessions per week. Among the included studies, three employed active controls while 30 used passive controls; 15 studies implemented high-cognition-targeted PA, whereas 19 adopted low-cognition-targeted PA. Additionally, 10 studies implemented game-based interventions in the experimental groups. Regarding outcome measures, CF, IC, and WM were evaluated in 22, 31, and 22 studies, respectively. Detailed characteristics of the included studies are summarized in Table 1.

**Table 1:** Studies included for meta-analysis.

Study/Country	Age (Years)	Total sample SIZE	Time/frequency/duration	Intervention protocol		Outcomes		
				Experiment	Control	CF	IC	WM
Jiang and Zeng (2015) China	5 ~ 6	61	35 min/2 times/week 8 week	Moderate intensity football	Blank	FIS	Panda/Lion and rass/snow	CBT and RCBT
Wang C. et al. (2024) China	5 ~ 6	73	30 min/2 times/week 5 week	Music intervention	Daily activities	/	Day-Night Stroop	/
Zhang et al. (2024) China	5 ~ 6	63	60 min/3 times/week 12 week	Gymnastics	Watched cartoons	DCCS	Flanker	1-back
Hou et al. (2023) China	4	113	30 min/2 times/week 8 week	Sports games based on displacement movement skills	free Movement	DCCS	Fish-Shark	Mr. Ant
Frischen et al. (2019) Germany	5 ~ 6	96	20 min/3 times/week 20 week	Group a: rhythm training Group b: exercise training	Pitch training	DCCS	“Statue” subtest	CBT
Giordano and Alesi (2022) Italy	5 ~ 6	50	20 min/3 times/week 6 week	Cognitively engaging PA	Blank	/	Day-Night Stroop	/
Mavilidi et al. (2023) Australia	3 ~ 4	73	15 min/2 times/week 6 week	cognitively engaging PA	reading	DCCS	Go/No-Go	Mr. Ant
Mulvey et al. (2018) America	3 ~ 6	107	30 min/2 times/week 6 week	SKIP (a gross motor intervention)	Free movement	/	HTKS	/
Wang S. et al. (2024) China	5 ~ 6	62	30–40 min/3 times/week 6 week	Rhythmic-movement activity	Blank	/	GDT	/
Miller et al. (2023) America	3 ~ 5	112	45 min/3 times/week 16 week	CHAMP	Free movement	Boats and Rabbits	HTKS	Mr. Ant
Franziska et al. (2022) Germany	5 ~ 6	25	20 min/3 times/week 14 week	Sports program	Music program	/	“Statue” subtest	/
Lai et al. (2020) China	6	20	60 min/2 times/week 8 week	Tennis games	Routine classroom activities	/	/	1-back
Wang et al. (2018) China	5 ~ 6	63	45 min/2 times/week 16 week	Taekwondo	Routine classroom activities	DCCS	Flanker	Sequence memory
Wen et al. (2018) China	3 ~ 5	57	20 min/5 times/week 10 week	The mini-trampoline PA intervention	Blank	FIS	Go/No-Go	WMS
Bai et al. (2022) China	4 ~ 5	62	50 min/3 times/week 8 week	Cognitive-demanding MVPA games	Routine activities	DCCS	SSS	Empty house
Jiao et al. (2020) China	4 ~ 5	51	30 min/3 times/week 5 week	Group games of body movements	Watched cartoons or free play	FIS	Day-Night Stroop	Self-Ordered Pointing
Wang H. et al. (2022) China	4 ~ 5	71	40 min/4 times/week 12 week	Pom cheerleading	Troutine exercises	FIS	Panda/Lion	CBT
Kosokabe et al. (2021) Japan	4 ~ 5	167	30 min/5 times/week 6 week	Music play program	Singing in unison or picture-book reading	DCCS	black white	BDS
Liu et al. (2022) China	4 ~ 5	48	30 min/5 times/week 4 week	Exergames	Conventional PA	DCCS	Go/No-Go	Mr. Ant
Jarraya et al. (2022) Germany	5 ~ 6	52	30 min/2 times/week 12 week	Group a: yoga Group b: kindergarten-based PMR	Blank	/	“statue” subtest	/
Xiong et al. (2019) China	4 ~ 5	60	20 min/5 times/week 8 week	Exergames	Traditional PA program	DCCS	/	/
Shen et al. (2020). China	3 ~ 6	60	40–50 min/3 times/week 8 week	street dance training	free movement	DCCS	Go/No-Go	BDS
Shen et al. (2019) China	4	61	45 min/5 times/week 12 week	music training	blank	DCCS	Day-Night Stroop	BDS
Gao et al. (2019) China	4 ~ 6	32	30 min/5 times/week 12 week	educational exergames	regular exercises	DCCS	/	/
Schmidt et al. (2020) Switzerland	4 ~ 6	137	15 min/4 times/week 6 week	physical-cognitive training program	blank	DCCS	Day-Night Stroop	N-back
Li et al. (2025) China	5 ~ 6	88	30 min/2 times/week 10 week	Group a: martial arts sensory teaching Group b: martial arts traditional teaching	free activity	/	flanker	/
Ltifi et al. (2025) Tunisia	3 ~ 4	54	20 min/3 times/week 12 week	mini-trampoline training	preschool PA	/	Go/No-Go	Mr. Ant
Başarır et al. (2025) Türkiye	5 ~ 6	51	25 min/2 times/week 8 week	coordination-based training	regularly scheduled lessons	/	Go/No-Go	/
Liu et al. (2025) China	4 ~ 6	201	45 min/2 times/week 8 week	Early childhood dancesport intervention	Regular activities	DCCS	Flanker	BDST
Legarra-Gorgoñon et al. (2025) Spain	5	80	16-32 min/2 times/week 12 week	Gamified training	Routine care	DCCS	Go/No-Go	Mr. Ant
Hao et al. (2025) China	4 ~ 6	80	45 min/2 times/week 12 week	Structured motor learning sessions	Regular outdoor free play	STS	ARR	HSE

FIS, Flexible Item Selection Task; CBT, The Corsi Blocks Test; RCBT, The Re-verse Corsi Blocks Test; DCCS, Dimensional Change Card Sort; HTKS, Head-Toes-Knees-Shoulders Task; GDT, Gift Delay Task; CHAMP, Children’ s Health Activity Motor Program; WMS, Working Memory Span; SSS, Silly Sound Stroop; BDS, Backward Digit Span; PMR, Progressive Muscle Relaxation; STS, Same Things Task; ARR, Arrows task; HSE, House task.

### 3.3 Methodological quality assessment

**Figure 2:**
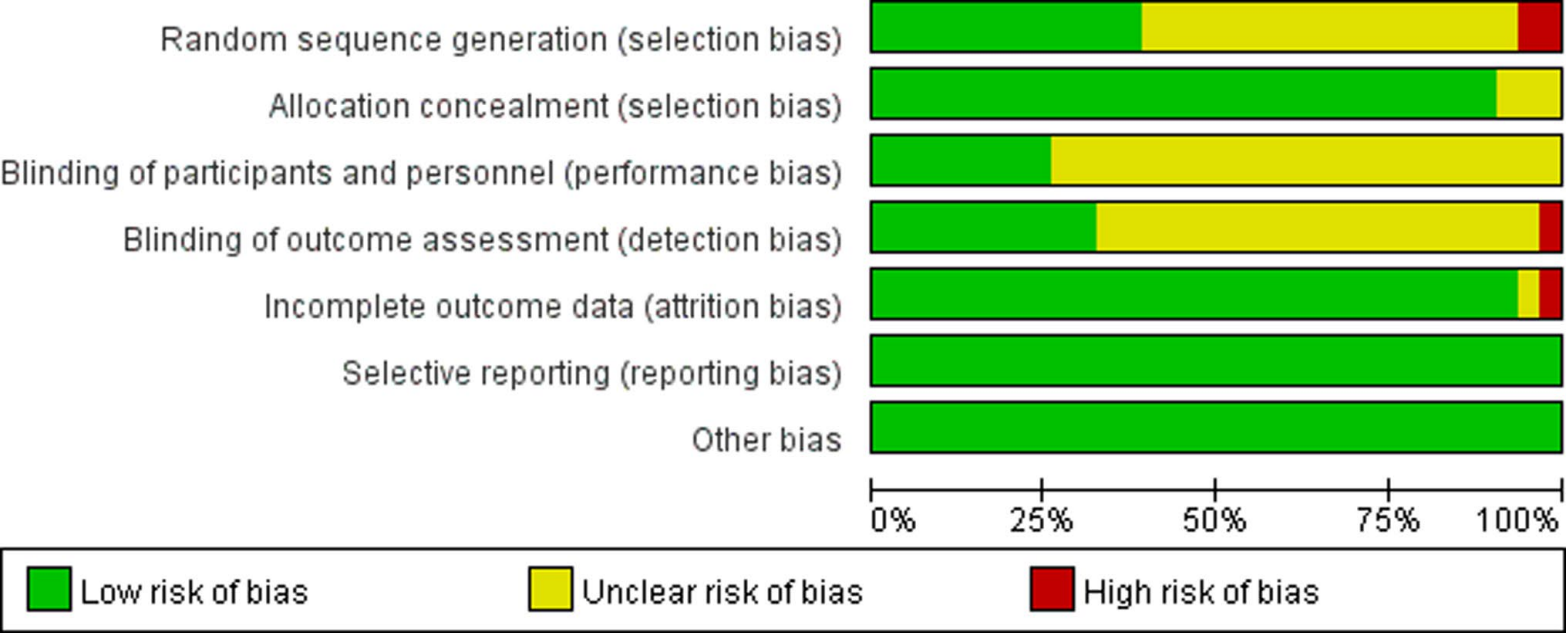
Risk of bias assessment results of included studies.

**Figure 3:**
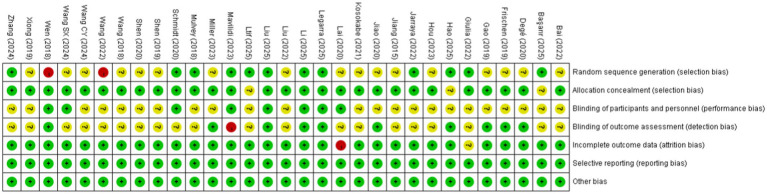
Percentage of biased items included.

We performed a Cochrane risk of bias assessment for each study, 16 studies were rated as high quality and 15 as moderate quality, with no studies classified as low quality. These findings indicate that all included studies demonstrated acceptable methodological rigor. The methodological quality of each individual study is presented in Figures 2, 3.

### 3.4 Main effect test

The heterogeneity tests revealed significant variation in the effects of PA on each dimension of EFs in preschool children (Q test, *p* < 0.001; *I*^2^ > 50% for all domains), indicating substantial heterogeneity across effect sizes. Accordingly, a random-effects model was applied in the subsequent analyses.

**Figure 4:**
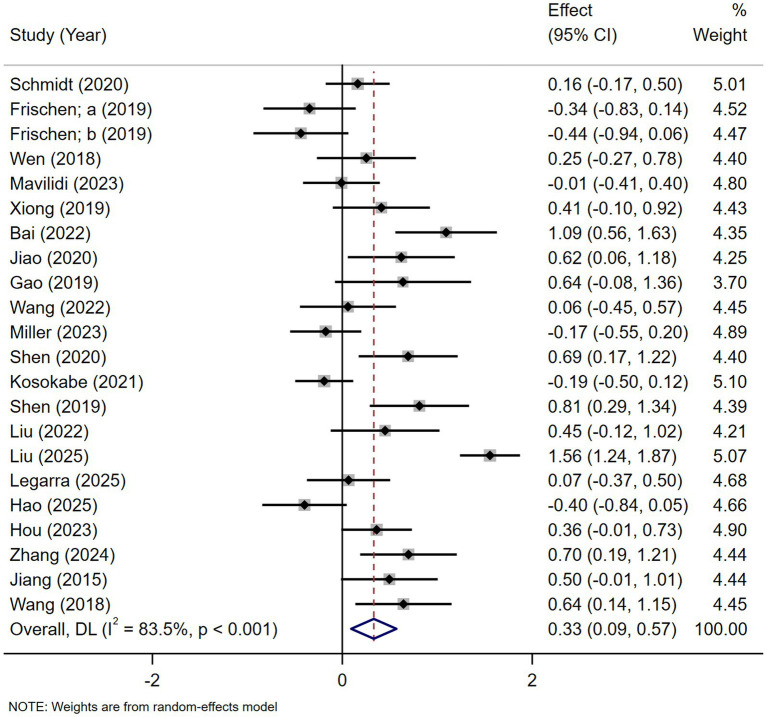
Forest plot showing the effects of PA vs. control on CF.

**Figure 5:**
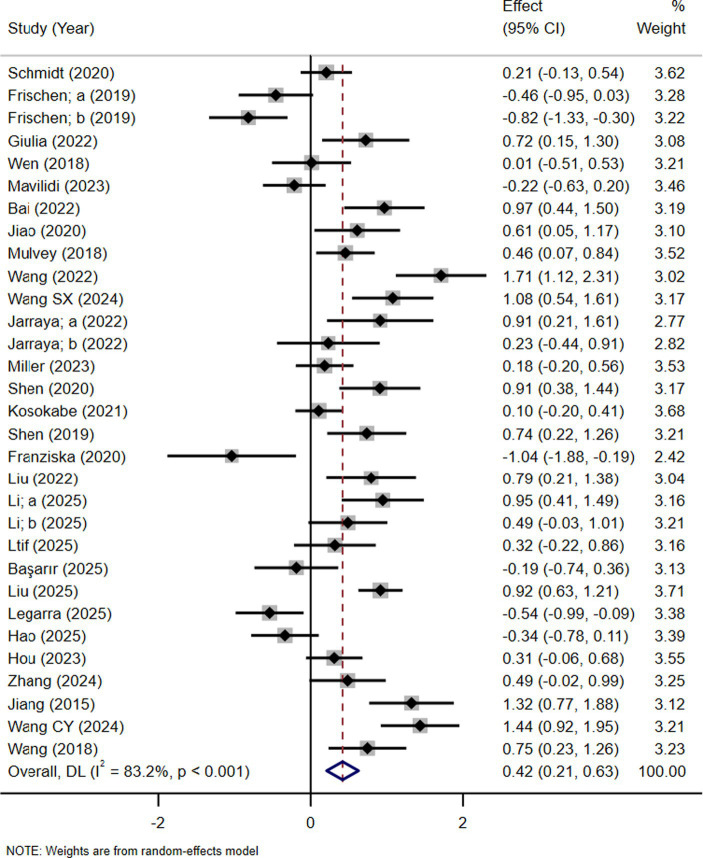
Forest plot showing the effects of PA vs. control on IC.

**Figure 6:**
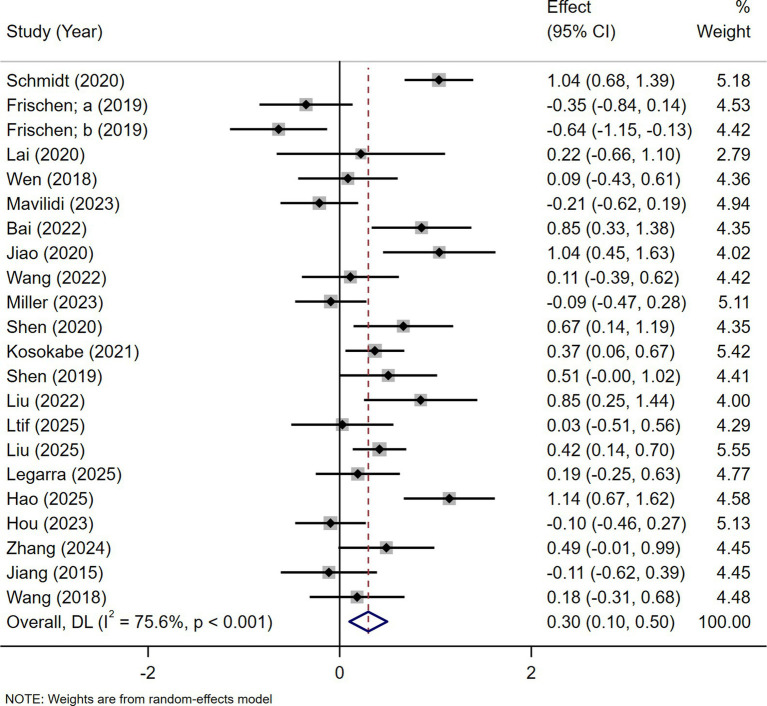
Forest plot showing the effects of PA vs. control on WM.

The overall effects of PA on preschoolers’ EFs are shown in Figures 4–6. Initial pooled analyses including all identified studies showed that PA had significant positive effects on CF (SMD = 0.33, 95% CI [0.09, 0.57], *I*^2^ = 83.5%, *p* < 0.001), IC (SMD = 0.42, 95% CI [0.21, 0.63], *I*^2^ = 83.2%, *p* < 0.001), and WM (SMD = 0.30, 95% CI [0.10, 0.50], *I*^2^ = 75.6%, *p* < 0.001). Overall, these results suggest that PA exerts small-to-moderate benefits across all three EF domains, with the largest effect observed for IC, followed by CF and WM.

**Figure 7:**
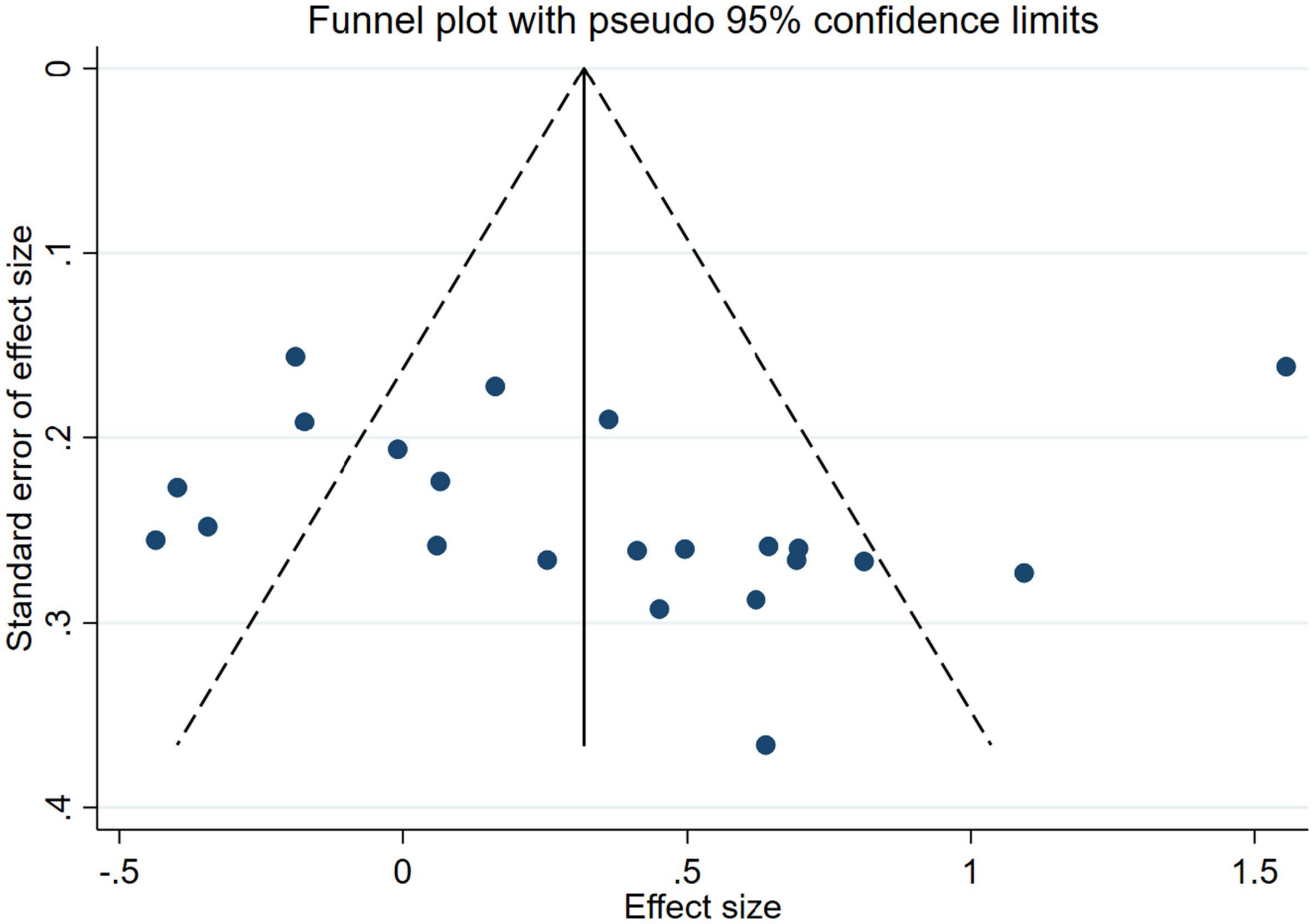
The funnel plot of publication bias for CF.

**Figure 8:**
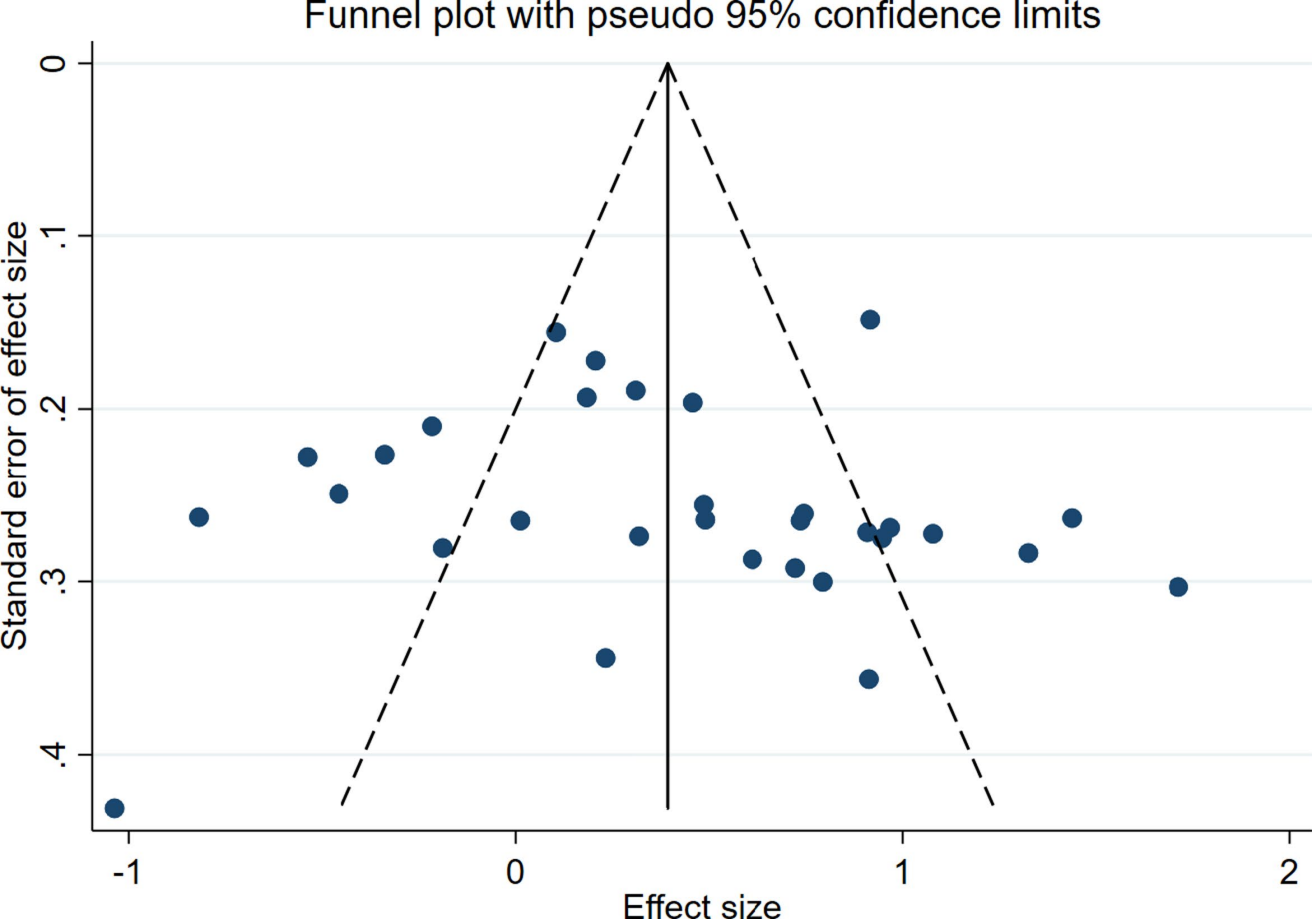
The funnel plot of publication bias for IC.

**Figure 9:**
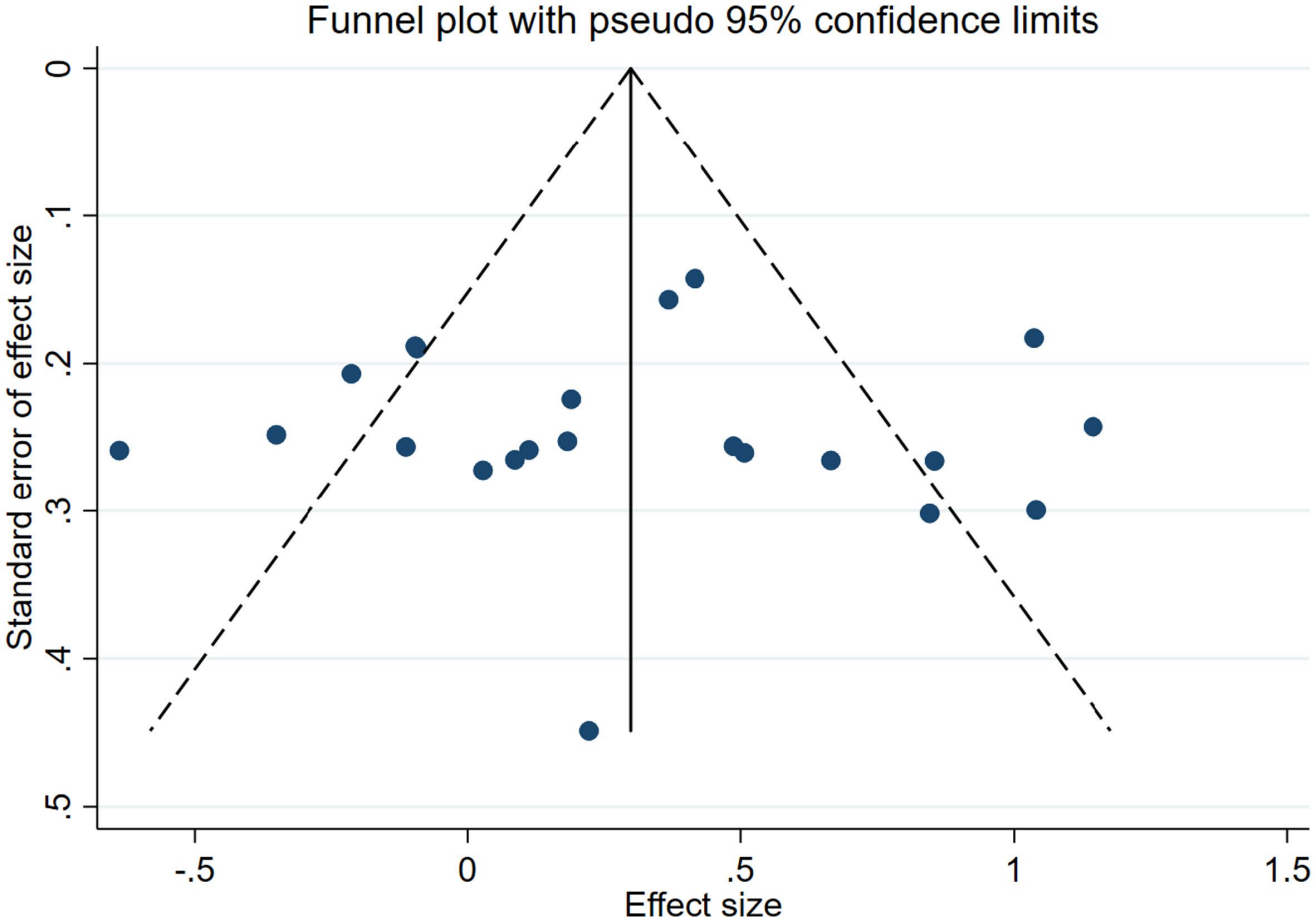
The funnel plot of publication bias for WM.

Figures 7–9 present funnel plots for CF, IC, and WM, respectively. Egger’ s test showed no significant publication bias for CF (*p* = 0.800), IC (*p* = 0.558), or WM (*p* = 0.986).

**Figure 10:**
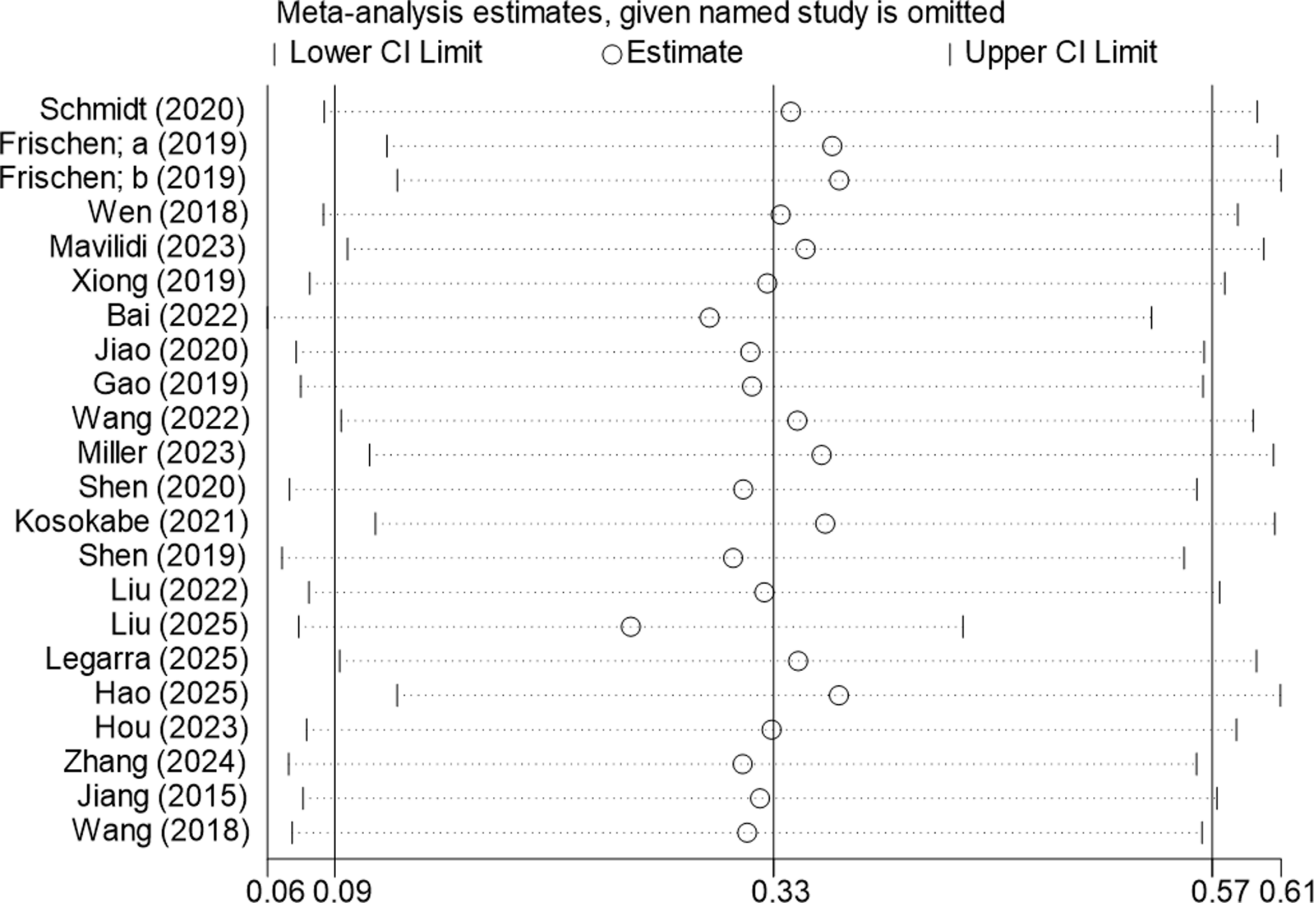
The influence of each study on the outcome of the meta-analysis(CF).

**Figure 11:**
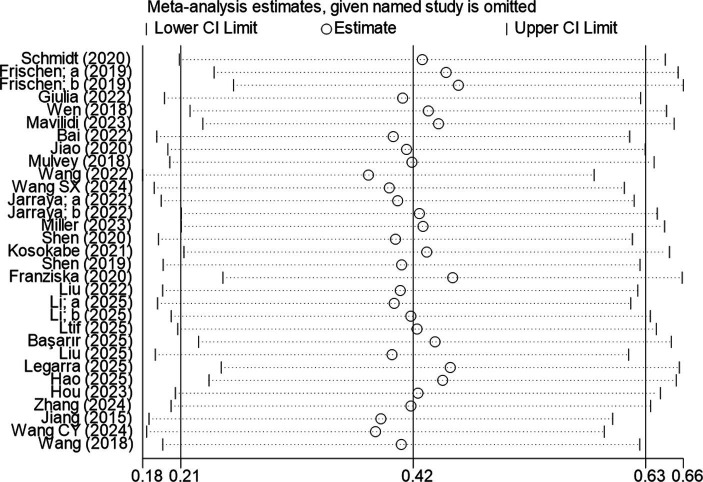
The influence of each study on the outcome of the meta-analysis(IC).

**Figure 12:**
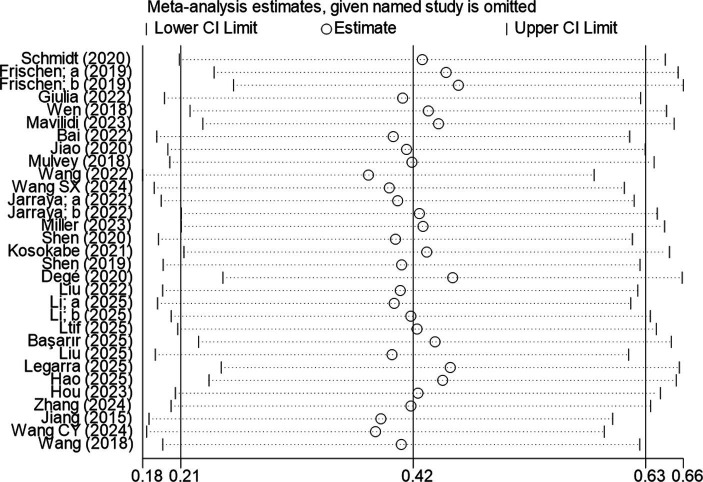
The influence of each study on the outcome of the meta-analysis(WM).

Sensitivity analyses were conducted by sequentially excluding each included study. The pooled effect sizes remained statistically significant, suggesting that the findings of this meta-analysis are relatively robust (Figures 10–12).

Funnel plots revealed that Liu et al. (2025) and Franziska et al. (2022) deviated from the primary cluster of studies, indicating that they may serve as outliers that either overestimate or underestimate the true effect. Sensitivity analyses corroborated this finding: the exclusion of Liu et al. (2025) from the CF domain decreased the pooled effect size from SMD = 0.33 to 0.25 and reduced heterogeneity from *I*^2^ = 83.5 to 68.2%, signifying that it was a significant source of heterogeneity and warranted exclusion from the final analysis. In contrast, the removal of Franziska et al. (2022) from the IC domain had a negligible effect on the pooled effect (SMD = 0.42 to 0.45) and on heterogeneity (*I*^2^ = 83.2 to 82.6%), suggesting that it exerted limited influence on the overall results; consequently, it was retained.

### 3.5 Moderator analysis

Given the substantial heterogeneity observed across effect sizes, subgroup analyses were conducted to explore potential sources of variability. The moderators included cognition-targeted interventions, game-based interventions, type of control, intervention duration, frequency, and session time (Table 2).

**Table 2:** Analysis of moderating variables of the effect of PA on young children’ s CF, IC, andWM.

EFs	Classification	Moderator	Level	Number of trials	Sub-analysis			Between-group homogeneity		
					SMD	95%CI	*I*^2^%	*Q*-value	Df (*Q*)	*p*-value
CF	Intervention type	Cognition targeted	High	9	0.26	[−0.06, 0.57]	80%	0	1	0.986
			Low	12	0.26	[0.05, 0.47]	52%			
		Game-based interventions	Game-based	7	0.54	[0.28, 0.80]	45%	6.50	1	0.011*
			Non-game-based	14	0.11	[−0.09, 0.32]	65%			
		Type of control	Active	3	−0.13	[−0.65, 0.39]	69%	2.44	1	0.118
			Passive	18	0.31	[0.13, 0.50]	66%			
	Intervention time	Duration	≤6 week	5	0.14	[−0.13, 0.41]	53%	7.24	2	0.027*
			>6 to <10 week	5	0.58	[0.33, 0.83]	30%			
			≥10 week	11	0.14	[−0.13, 0.42]	71%			
		Session time	<30 min	7	0.02	[−0.19, 0.23]	34%	6.62	2	0.036*
			≥30 to <45 min	8	0.21	[−0.07, 0.49]	64%			
			≥45 min	6	0.61	[0.21,1.01]	75%			
		Frequency	<3 times	6	0.18	[−0.11, 0.47]	62%	0.27	2	0.874
			≥3 to <4 times	7	0.29	[−0.15, 0.74]	83%			
			≥4 times	8	0.27	[0.03, 0.51]	53%			
IC	Intervention type	Cognition Targeted	High	15	0.56	[0.31, 0.81]	81%	1.74	1	0.187
			Low	16	0.27	[−0.07, 0.62]	85%			
		Game-based interventions	Game-based	8	0.47	[0.07, 0.87]	83%	0.08	1	0.773
			Non-game-based	23	0.40	[0.15, 0.65]	83%			
		Type of control	Active	3	−0.69	[−1.02,-0.36]	0%	39.22	1	<0.001**
			Passive	28	0.53	[0.33, 0.73]	79%			
	Intervention time	Duration	≤6 week	9	0.55	[0.22, 0.87]	79%	3.68	2	0.158
			>6 to <10 week	6	0.70	[0.31,1.10]	78%			
			≥10 week	16	0.23	[−0.10, 0.56]	84%			
		Session time	<30 min	10	−0.17	[−0.47, 0.12]	72%	23.92	2	<0.001**
			≥30 to <45 min	14	0.70	[0.40,1.00]	81%			
			≥45 min	7	0.70	[0.46, 0.93]	48%			
		Frequency	<3 times	14	0.45	[0.13, 0.78]	85%	0.84	2	0.657
			≥3 to <4 times	11	0.29	[−0.10, 0.68]	84%			
			≥4 times	6	0.56	[0.11,1.00]	83%			
WM	Intervention type	Cognition Targeted	High	10	0.55	[0.27, 0.84]	79%	7.52	1	0.006*
			Low	12	0.06	[−0.15, 0.26]	49%			
		Game-based interventions	Game-based	7	0.42	[0.14, 0.71]	62%	0.89	1	0.346
			Non-game-based	15	0.24	[−0.02, 0.50]	80%			
		Type of control	Active	2	−0.49	[−0.84,-0.14]	0%	18.09	1	<0.001**
			Passive	20	0.38	[0.19, 0.56]	71%			
	Intervention time	Duration	≤6 week	5	0.60	[0.11,1.08]	84%	2.57	2	0.277
			>6 to <10 week	6	0.32	[0.00, 0.63]	64%			
			≥10 week	11	0.15	[−0.12, 0.42]	72%			
		Session time	<30 min	7	0.03	[−0.41, 0.48]	85%	2.36	2	0.307
			≥30 to <45 min	7	0.45	[0.07, 0.83]	79%			
			≥45 min	8	0.39	[0.18, 0.60]	40%			
		Frequency	<3 times	8	0.21	[−0.08, 0.51]	72%	1.89	2	0.388
			≥3 to <4 times	8	0.24	[−0.16, 0.64]	81%			
			≥4 times	6	0.50	[0.18, 0.82]	67%			

The subgroup analysis for intervention type revealed several significant moderating effects. For CF, game-based interventions showed a significant and moderate improvement (SMD = 0.54, 95% CI [0.28, 0.80], *I*^2^ = 45%), whereas non-game-based interventions did not yield a significant effect (SMD = 0.11, 95% CI [−0.09, 0.32]), with a statistically significant between-group difference (*p* = 0.011). In contrast, the level of cognitive engagement (cognition targeted) was not a significant moderator for CF (*p* = 0.986) or for IC (*p* = 0.187). However, for WM, higher cognitive engagement was associated with a significant and moderate benefit (SMD = 0.55, 95% CI [0.27, 0.84]), and the subgroup difference was significant (*p* = 0.006). The type of control group also significantly moderated the effects on IC and WM. For IC, passive control groups showed a moderate beneficial effect (SMD = 0.53, 95% CI [0.33, 0.73]), while active control groups were associated with a significant negative effect (SMD = −0.69, 95% CI [−1.02, −0.36]), resulting in a highly significant subgroup difference (*p* < 0.001). A similar pattern was observed for WM, where passive control groups demonstrated a significant positive effect (SMD = 0.38, 95% CI [0.19, 0.56]), in contrast to active control groups, which showed a negative effect (SMD = −0.49, 95% CI [−0.84, −0.14]); the between-group difference was also highly significant (*p* < 0.001).

The subgroup analysis for intervention time also revealed several significant moderating effects. Intervention duration significantly moderated the effect on CF (*p* = 0.027). Interventions lasting between >6 and <10 weeks produced a moderate and significant improvement (SMD = 0.58, 95% CI [0.33, 0.83]), while shorter (≤6 weeks) or longer (≥10 weeks) interventions did not show significant effects. Session time was also a significant moderator for both CF and IC. For CF, sessions lasting ≥45 min had a large and significant effect (SMD = 0.61, 95% CI [0.21, 1.01]), which was significantly different from shorter sessions (*p* = 0.036). For IC, both sessions of ≥40 to <45 min and ≥45 min showed moderate-to-large significant effects (SMD = 0.70 for both), while sessions <30 min showed no significant effect, with a highly significant subgroup difference (*p* < 0.001). Other potential moderators did not exhibit significant subgroup differences. Frequency of intervention sessions did not significantly moderate effects for any executive function: CF (*p* = 0.874), IC (*p* = 0.657), or WM (*p* = 0.388). Similarly, intervention duration was not a significant moderator for IC (*p* = 0.158) or WM (*p* = 0.277), and session time was not significant for WM (*p* = 0.307).

**Figure 13:**
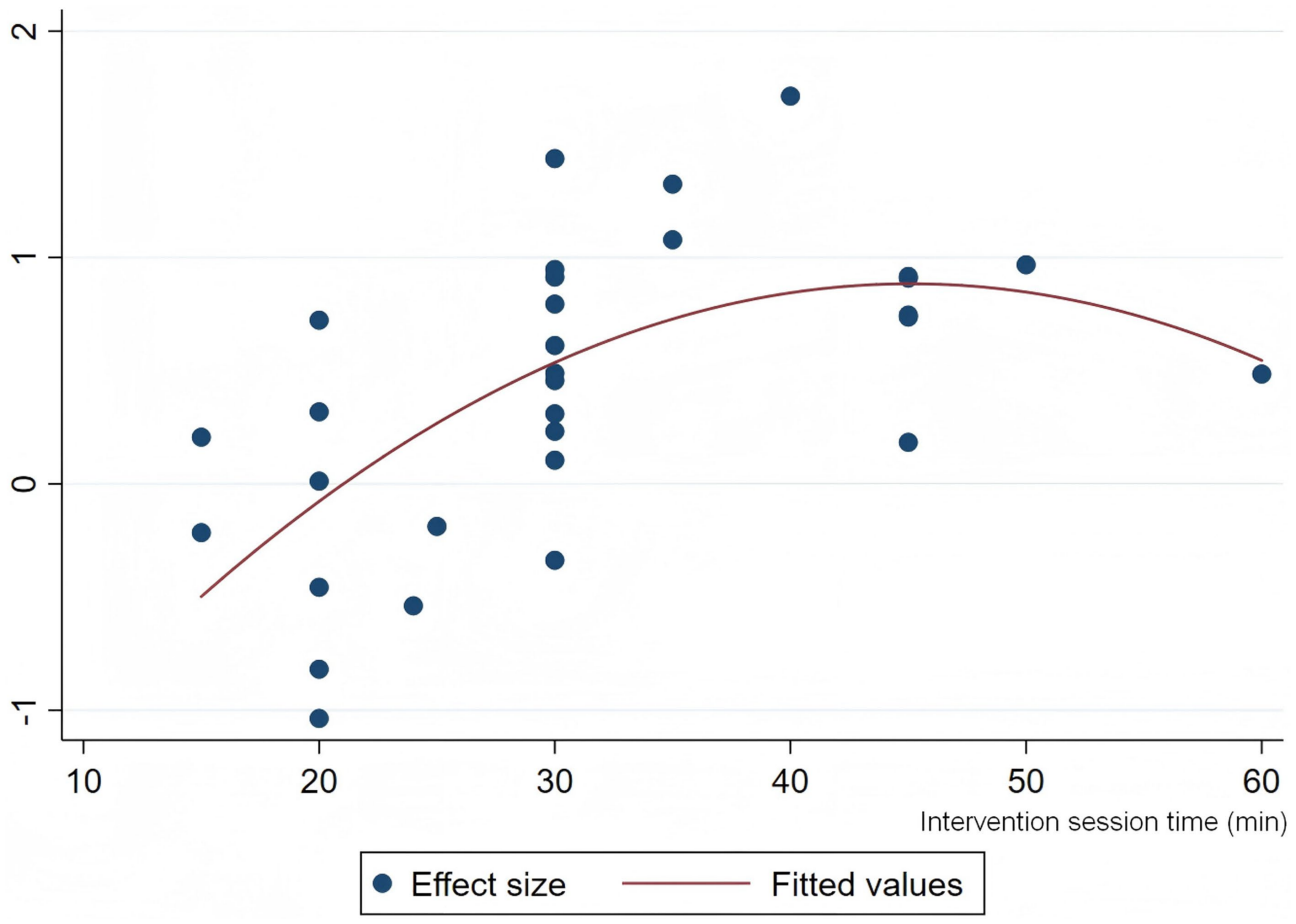
Effect size by intervention session time in meta-regression for IC.

For continuous variables identified as significant in subgroup analyses, meta-regression was performed to further examine their associations with effect sizes. Quadratic meta-regression revealed a significant non-linear relationship between session time and effect size for IC (*F*(2, 28) = 8.32, *p* = 0.0015), with a negative quadratic coefficient (*β* = −0.0014, 95% CI [−0.0027, −0.00004], *p* = 0.044), indicating an inverted U-shaped pattern (Figure 13). The model explained 39.08% of between-study heterogeneity (adjusted R^2^), outperforming the linear model (31.10%). The estimated peak effect occurred at approximately 46 min per session (95% CI [42, 50]), after which the effect gradually declined, suggesting an optimal session duration rather than a simple “longer is better” relationship.

**Figure 14:**
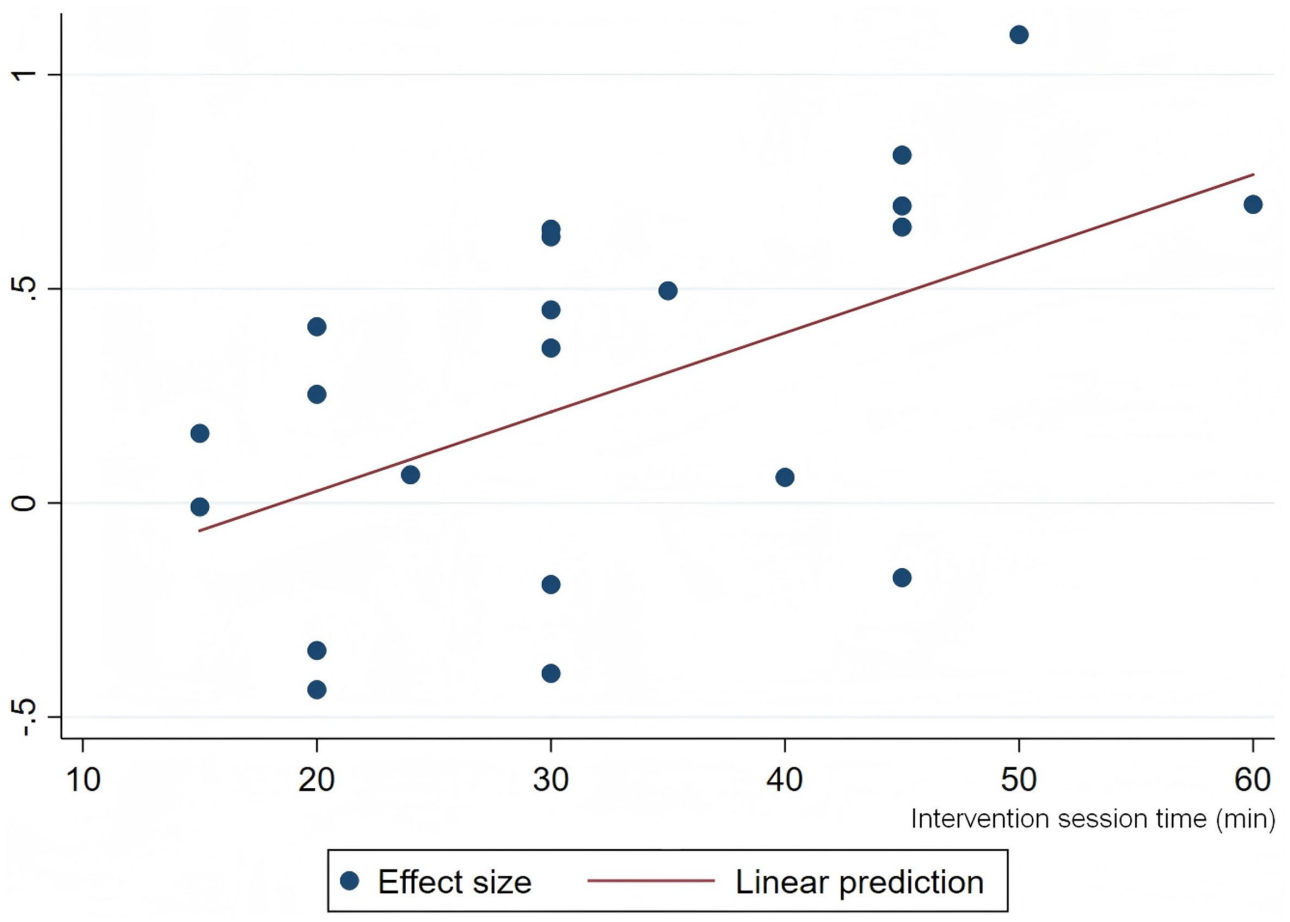
Effect size by intervention session time in meta-regression for CF.

For CF, linear meta-regression indicated a significant positive relationship between session time and effect size (β = 0.018, 95% CI [0.004, 0.033], *p* = 0.013) (Figure 14), explaining 32.89% of heterogeneity. Each additional unit of session time corresponded to an increase of 0.018 in effect size.

Although subgroup analyses suggested an absolute advantage for 8-week CF interventions, meta-regression did not reveal a statistically significant association between intervention duration and effect size. These results provide valuable guidance for future dose–response studies.

## 4 Discussion

This meta-analysis confirmed that PA significantly enhances all three core dimensions of EFs in preschool children—CF, IC, and WM—though effect sizes varied across domains. The main effect analyses indicated small-to-moderate improvements, with IC showing the largest gain (SMD = 0.42), followed by WM (SMD = 0.30) and CF (SMD = 0.25). These cognitive benefits may be supported by underlying neurobiological mechanisms, as PA has been posited to promote EFs development through the modulation of neurotransmitter levels and the enhancement of neural plasticity (Du et al., 2024). These results are consistent with the large-scale review by Wei et al. (2024) and comparable in effect size to the studies by Chen et al. (2023), collectively supporting the broad benefits of PA for EFs development in young children (Wei et al., 2024). Substantial heterogeneity across studies (*I*^2^ > 65%) highlights differences in intervention protocols and underscores the need for subsequent moderator analyses.

Notably, one study by Liu et al. (2025) investigating the effect of DanceSport on EFs was excluded from the CF analysis due to high risk of bias and substantial heterogeneity. This study reported unusually large improvements in CF among 4-6-year-olds following an 8-week DanceSport intervention, which may be attributed to its cognitively demanding design, specific age-sensitive and game engaged intervention components. The exclusion of this outlier helps ensure the robustness and generalizability of the present meta-analytic findings, as its inclusion would have disproportionately inflated the overall effect size for CF and obscured the more modest yet consistent effects observed across other included studies.

Regarding intervention type, cognition-targeted PA was associated with a significant moderating effect on WM. Previous studies have suggested that cognitively engaging PA may enhance cognitive function more effectively than non-cognitive PA, such as repetitive exercises (Best, 2012; Schmidt et al., 2020). In recent years, cognitively engaging PA interventions have gained increasing attention. These interventions integrate cognitive challenges—such as rule-switching, IC, and WM updating—into movement contexts, concurrently engaging motor and cognitive systems (Preston et al., 2025; Xu et al., 2025). A recent meta-analysis by Pacheco et al. (2025) reported that interventions incorporating cognitively engaging activities significantly improved all three EFs subdomains and attention in preschool children, showing greater benefits than general PA programs.

Game-based interventions also appeared to confer advantages for enhancing CF, consistent with previous research indicating that game-based PA can significantly improve CF in young children(Chaddock-Heyman et al., 2013). This finding is in line with both Piaget’s view of play as a manifestation of cognition (Pakpahan and Saragih, 2022; Veraksa et al., 2022) and the embodied cognition perspective on physical play (Serino and Ossmy, 2025). Several studies consistently highlight the superior effects of gamified PA interventions(Chen et al., 2023). These effects may be attributed to several characteristics of such activities. Firstly, their developmental appropriateness and multitask structure support the coordinated development of WM and cognitive control (Cornejo et al., 2021). Secondly, the “safe failure” environment which games provide aligns with a dialectical learning model, allowing children to build adaptive responses through trial and error—a direct manifestation of CF (Diamond, 2013; Veraksa et al., 2022). Finally, play-based PA emphasis on peer interaction and social cooperation helps integrate motor and social development, while fostering positive relationships that are critical for enhancing EFs (Hamre et al., 2014; Holmes et al., 2016).

Control type exhibited a significant moderating effect for IC and WM (*p* ≤ 0.001), with passive control groups consistently showing greater intervention effects than active control groups. On one hand, when the control group also engages in some form of activity (active control), the observed effect of PA interventions may be underestimated because the control condition itself provides cognitive or physical stimulation. On the other hand, passive control groups (e.g., blank controls, free play) may lead to overestimation of PA effects, as they do not control for non-specific factors such as social interaction, adult attention, novelty, or expectancy effects. Accordingly, careful consideration of control group design is recommended to ensure accurate assessment of intervention efficacy.

Regarding intervention time, meta-regression revealed a significant positive linear relationship between session duration and CF effect size, while a nonlinear (inverted U-shaped) relationship was identified for IC, with effect sizes peaking around 46 min before declining. Given that IC is supported by the prefrontal cortex (PFC), these patterns have been discussed in relation to increased cerebral blood flow in the PFC during prolonged PA (Wang C. et al., 2022). This pattern is consistent with Lu et al. (2025), whose meta-analysis of music training also found that sessions exceeding 40 min yielded greater benefits for CF. Furthermore, Lu et al. reported that WM responded better to shorter sessions of 20–30 min, suggesting different EFs may exhibit distinct sensitivities to intervention duration.

Although interventions around 8 weeks showed the largest effect sizes for CF in subgroup analyses, this duration did not reach statistical significance in any meta-regression models. This discrepancy may be attributed to limited statistical power in the regression models, the apparent advantage of an 8-week duration might be confounded by other unmeasured study characteristics, such as intervention modality or participant demographics. Additionally, no significant moderating effects were observed for intervention frequency (*p* > 0.05), which aligns with previous meta-analyses by Li et al. (2020), Song H. et al. (2023), and Morales et al. (2024).

Taken together, these findings highlight the potential of PA to enhance EFs in preschool children, while also underscoring the specific influence of various intervention characteristics and moderators on different EF domains. These results may be informative for understanding how PA interventions relate to EF outcomes in early childhood settings. Current evidence suggests that PA interventions in preschool settings should prioritize sessions approximately 45 min, incorporate gamified elements, and include progressive cognitive challenges, particularly when aiming to improve WM and CF. Furthermore, while the optimal intervention duration warrants further confirmation, our findings cautiously suggest that an intervention period of approximately 8 weeks may be associated with CF outcomes within a single intervention program.

Considering the developmental trajectories and neurobiological bases of different EF components, future research should focus on several key directions: (1) developing targeted PA-cognition combined interventions specifically for WM; (2) employing multimodal assessments (e.g., fMRI combined with behavioral testing) to investigate underlying neural mechanisms; and (3) conducting long-term follow-ups to examine the sustainability of intervention effects. Pursuing these directions will contribute to the design of more precise PA interventions to promote cognitive development, thereby helping to refine the understanding of PA–EF relationships in early childhood.

## 5 Limitations of the study

Several limitations of this study should be acknowledged. First, only studies published in Chinese and English were included, which may introduce language bias. Second, although this meta-analysis systematically integrated various types of PA interventions to offer valuable guidance for practice, the wide variation in intervention protocols and assessment tools may have contributed to substantial methodological heterogeneity. While subgroup analyses were conducted to explore sources of heterogeneity, some subgroups had limited sample sizes, which may restrict the generalizability of the findings. Furthermore, while children aged 3–6 years show rapid cognitive development and substantial individual differences, the majority of studies failed to report stratified data by age or sex. Recent research has begun to highlight these age-dependent effects, particularly on specific EF subdomains (Deja et al., 2025; Liu et al., 2025). Consequently, information on baseline cognitive levels, training intensity, and other potential moderators was insufficient, limiting the depth of further analyses. Nevertheless, sensitivity analyses showed that sequentially removing individual studies did not substantially alter the results, indicating that the main conclusions remain relatively robust. Finally, some control groups engaged in daily PA, which may have led to an underestimation of intervention effects; however, this also reflects real-world educational settings and enhances the practical applicability of the findings.

## 6 Conclusion

This meta-analysis demonstrates that PA can enhance all three core EFs—CF, IC, and WM—in preschool children, with the most pronounced effects observed for IC. Moderator analyses indicate that intervention type and design significantly influence outcomes: game-based approaches are particularly effective for CF, while cognitively engaging activities benefit WM. In addition, sessions lasting approximately 45 min are associated with stronger effects, especially for CF and IC. The use of passive control groups also appears to amplify observed effect sizes for IC and WM compared to active control designs. Furthermore, our findings cautiously suggest that an 8-week period may represent a key node for fostering CF within a single intervention program. In contrast, intervention frequency did not consistently moderate the effects. Collectively, these findings support the integration of cognitively engaging, and game-based PA sessions into early childhood curricula, with attention to session length and cognitive demands. Future studies should adopt more rigorous designs, including active control groups and longitudinal follow-ups, to strengthen causal inference and clarify the sustained benefits of PA on EFs in young children.

## Data Availability

The original contributions presented in the study are included in the article/Supplementary material, further inquiries can be directed to the corresponding author.
