# Supplementary material for: The effects of physical activity on executive function in preschool children: a meta-analysis of randomized controlled trials
Source: Front Psychol. 2026 Jul 17;17:1882118. doi: 10.3389/fpsyg.2026.1882118 (PMC13423684; doi:10.3389/fpsyg.2026.1882118)
Supplement: Supplementary file 1 [file Table_1.doc]

**Search strategy**

**China national knowledge infrastructure 431**

SU%= (‘学龄前’+'学前儿童'+‘幼儿’)*(‘认知功能’+‘执行功能’+‘认知灵活性’+‘抑制控制’+‘工作记忆’+‘抑制’+‘刷新’+‘转换’)*(‘身体活动’+‘动作’+‘锻炼’+‘体育’+‘游戏’+‘运动’+‘体适能’+‘音乐’+‘舞蹈’)

**Wanfang data 1112**

主题:(“学龄前”OR”学前儿童”OR”幼儿”)AND(“认知功能”OR”执行功能”OR”认知灵活性”OR”抑制控制”OR”工作记忆”OR”抑制”OR”刷新”OR”转换”)AND(“身体活动”OR”动作”OR”锻炼”OR”体育”OR”游戏”OR”运动”OR”体适能”OR”音乐”OR”舞蹈”)

**VIP databases 726**

(M=( 学龄前 + 学前儿童 + 幼儿 )*( 认知功能 + 执行功能 + 认知灵活性 + 抑制控制 + 工作记忆 + 抑制 + 刷新 + 转换 )*( 身体活动 + 动作 + 锻炼 + 体育 + 游戏 + 运动 + 体适能 + 音乐 + 舞蹈 ) OR R=( 学龄前 + 学前儿童 + 幼儿 )*( 认知功能 + 执行功能 + 认知灵活性 + 抑制控制 + 工作记忆 + 抑制 + 刷新 + 转换 )*( 身体活动 + 动作 + 锻炼 + 体育 + 游戏 + 运动 + 体适能 + 音乐 + 舞蹈 ) )

**Web of Science Core Collection 3170**

TS=(physical activity OR activity OR physical education OR exercise OR sport OR active games OR active play OR physical fitness OR music OR dance) AND TS=(kindergarten OR Preschool OR young children OR early childhood) AND TS=(executive function OR working memory OR inhibitory control OR cognitive flexibility OR cognition OR cognitive function)

# **Cochrane Library 1234**

1：physical activity OR activity OR physical education OR exercise OR sport OR active games OR active play OR physical fitness OR music OR dance

2：kindergarten OR Preschool OR young children OR early childhood

3：executive function OR working memory OR inhibitory control OR cognitive flexibility OR cognition OR cognitive function

4：#1 AND #2 AND #3

**the EBSCO self-built database 546**

SU ((physical activity OR activity OR physical education OR exercise OR sport OR active games OR active play OR physical fitness OR music OR dance)) AND SU ((kindergarten OR Preschool OR young children OR early childhood)) AND SU ((executive function OR working memory OR inhibitory control OR cognitive flexibility OR cognition OR cognitive function))

**PubMed 836**

((physical activity[MeSH Terms] OR physical activity[Title/Abstract] OR activity[Title/Abstract] OR physical education[Title/Abstract] OR exercise[Title/Abstract] OR sport[Title/Abstract] OR active games[Title/Abstract] OR active play[Title/Abstract] OR physical fitness[Title/Abstract] OR music[Title/Abstract] OR dance*[Title/Abstract]) AND (kindergarten[Title/Abstract] OR Preschool, child[MeSH Terms] OR Preschool[Title/Abstract] OR young children[Title/Abstract] OR early childhood[Title/Abstract]) AND (executive function[MeSH Terms] OR executive function[Title/Abstract] OR working memory[Title/Abstract] OR inhibitory control[Title/Abstract] OR cognitive flexibility[Title/Abstract] OR cognition[Title/Abstract] OR cognitive function[Title/Abstract]))
